# Adversity specificity and life period exposure on cognitive aging

**DOI:** 10.1038/s41598-023-35855-5

**Published:** 2023-05-29

**Authors:** M. Künzi, S. Sieber, E. Joly-Burra, S. Cullati, S. Bauermeister, S. Stringhini, B. Draganski, N. Ballhausen, M. Kliegel

**Affiliations:** 1 Dementias Platform UK, Department of Psychiatry, Warneford Hospital, University of Oxford, Oxford, OX3 7JX UK; 2 Center for the Interdisciplinary Study of Gerontology and Vulnerability (CIGEV), University of Geneva, Geneva, Switzerland; 3 LIVES, Overcoming Vulnerability: Life Course Perspective, Swiss National Centre of Competence in Research, Lausanne and Geneva, Switzerland; 4 Cognitive Aging Lab (CAL), University of Geneva, Geneva, Switzerland; 5 Population Health Laboratory (#PopHealthLab), University of Fribourg, Fribourg, Switzerland; 6 Department of Readaptation and Geriatrics, University of Geneva, Geneva, Switzerland; 7 Center for Primary Care and Public Health (Unisanté), University of Lausanne, Lausanne, Switzerland; 8 Unit of Population Epidemiology, Division of Primary Care, Geneva University Hospitals, Geneva, Switzerland; 9 Laboratory of Research in Neuroimaging (LREN), Department of Clinical Neuroscience, Lausanne University Hospital, University of Lausanne, Lausanne, Switzerland; 10 Neurology Department, Max Planck Institute for Human Cognitive and Brain Sciences, Leipzig, Germany; 11 Department of Developmental Psychology, Tilburg School of Social and Behavioral Sciences, Tilburg University, Tilburg, The Netherlands

**Keywords:** Psychology, Human behaviour

## Abstract

This study set out to examine the role of different adversities experienced at different life course stages on cognitive aging (i.e., level and change). Data from the longitudinal study: Survey of Health, Ageing, and Retirement in Europe (SHARE) with the selection of participants over 60 years were used (*N* = 2662, *Mdn*_*age*_ = 68, *SD*_*age*_ = 5.39) in a Structural Equation Modeling. In early life, the experience of hunger predicted lower delayed recall (β = − 0.10, *p* < 0.001) and verbal fluency (β = − 0.06, *p* = 0.001) performance in older age, whereas financial hardship predicted lower verbal fluency (β = − 0.06, *p* = 0.005) performance and steeper decline in delayed recall (β = − 0.11, *p* < 0.001). In early adulthood, financial hardship and stress predicted better delayed recall (financial hardship: β = 0.08, *p* = 0.001; stress: β = 0.07, *p* = 0.003) and verbal fluency performance (financial hardship: β = 0.08, *p* = 0.001; stress β = 0.10, *p* < 0.001), but no adversities were associated with a change in cognitive performance. In middle adulthood, no adversities were associated with the level of cognitive performance, but financial hardship predicted lower decline in delayed recall (β = 0.07, *p* = 0.048). This study highlights the importance of disentangling the period effect from the specific effect of the adversity experienced in the association between adversity and cognition in older age. Moreover, differential results for delayed recall and verbal fluency measures suggest that it is also important to consider the cognitive outcome domains examined.

## Introduction

The term adversity encompasses a wide variety of adverse experiences^1–3^ for which the specific adversity experienced, the occurrence (life course period at which the adversity has been experienced) but also the cumulative experiences with adversity are important and may have differential influence on brain and cognition^1,4,5^. In line with this assumption, available literature on adversity effects on cognitive aging has revealed contradictory findings preventing a global vision of the effects of adversity on cognition in aging to be constructed^6–10^. De facto, to achieve an overall picture of the effects of adversity on cognition in aging it seems key to consider both the specific adversity experienced and its occurrence (the associated life course period at which the adversity was experienced). Thus, the present study aims to disentangle the role of distinct adversities (stress, death of a parent, financial hardship, and hunger) experienced at three life course periods (early life, early adulthood, and middle adulthood) in predicting not only cognitive performance in older age but also change in cognitive performance across the aging process (level and change in cognition).

Why is it important to differentiate adverse experiences according to their occurrence in different life course periods? Studies on the effect of stress on the *brain* demonstrated that some stressors differently affect the brain depending on when in the life course they are experienced, suggesting the existence of sensitive periods^11,12^. Due to ongoing brain changes in specific brain regions (i.e., hippocampus, frontal cortex, and amygdala), some life course periods (particularly childhood, adolescence, and late-life) are more sensitive to the exposure to some stressors (e.g., trauma) than other life course periods, leading to long-term consequences (e.g., mental health, risky lifestyles)^1,12–17^. Not only the brain but also cognitive abilities in later life seem to be influenced by the life course period at which the adversity was experienced. Thus, in the following, results on associations between adversity and cognition will be reviewed by disentangling at which life course period they occurred.

### Lifecourse period of adversity and cognition

*Early/childhood* adverse circumstances or adverse socioeconomic position are found to be associated with later-life lower cognitive performance (i.e., global cognition, verbal learning, and verbal fluency^18^) including lower memory performance^18–20^. Interestingly, while disadvantageous socioeconomic conditions in childhood were associated with a lower level of delayed recall and verbal fluency in older age, they were also related to a slower decline in verbal fluency but not delayed recall^6^. However, this last result on cognitive change is inconsistent with the finding of Cermakova et al.^18^, where no association between socioeconomic position and change in cognition has been found. In contrast, some results suggest that the experience of some severe adversities (i.e., abuse) is associated with better cognitive performance (i.e., short-term memory performance, executive function, processing speed, and global cognitive function) and lower risk of cognitive impairment^8,21^.

Taken together, these studies emphasize the importance of the association between early / childhood experience of adversity and cognition in older age; yet, these associations seem to go in different directions depending on the specific adversity investigated.

In *young and middle adulthood* research investigating the effects of exposure to adversity on cognition and change in cognitive functions in older age has mainly focused on one specific adversity: disadvantageous socioeconomic conditions or status, such as low educational achievement and occupation^9,22–24^. Studies on socioeconomic conditions or status throughout the life course have shown that disadvantageous adulthood socioeconomic conditions (i.e., low levels or few years of education) were the strongest predictors of lower memory performance in older age compared to (indicators of) other life course stages^9,25^. Importantly, indicators of socioeconomic conditions are also used as proxy measures of cognitive reserve (e.g., education and occupation^26,27^) especially important in buffering cognitive impairment and early cognitive decline^28,29^.

Regarding the few studies focusing on adversity experienced in *late adulthood*, it has been found that older individuals who experienced an injury or an illness of a friend during the past years (and rating this event as having an impact on his/her life) have better episodic memory performance^10^. In relation to cognitive change, it has been shown that some stressful negative life events (e.g., death of (grand) child) are associated with a higher rate of cognitive decline whereas exposure to other stressors (e.g., illness of a partner) is associated with better cognition (i.e., less decline) in older adults^7^. High late-life income (an indicator of socioeconomic status) has been found to be the strongest predictor (compared to the childhood socioeconomic status composite score and education) of a slower decline in memory functioning in individuals aged over 50^25^. Furthermore, change in perceived stress (increased stress) has been found to be inversely associated with a change in short-term memory (decreased in immediate recall performance), but no association has been found between change in perceived stress and change in long-term memory and verbal fluency in participants aged 50 and older^30^.

### Distinct adversities and cognition

Although there appears to be an effect of the period of adversity exposure on brain and cognition, the effects found seem to differ depending on the adversity investigated. In addition, considering the broad range of distinct adversities that the adversity definition encompasses, it is essential to examine the differential effects of each adversity of interest (i.e., period of stress induced by an undefined stressor, parental death, disadvantageous socioeconomic conditions, or hunger) on cognition. Hence, the following paragraphs will focus on the differential associations of these specific adversities with the brain and cognition.

Studies on *stress* highlight that some stressors are specifically associated with effects on brain areas that are involved in learning, memory, and higher cognitive functions (i.e., hippocampus, amygdala, and (pre)frontal lobes^12,31,32^). Importantly, the effects of these stressors mainly depend on when in the life course they are experienced^12^. Nevertheless, some stressors may also enhance or impair cognitive function depending on the different stress levels (level of cortisol) they induced^7,12,33^.

Studies on the effect of *parental death* on cognition or dementia have found different results according to the life course period at which the loss was experienced but also depend on whether it is the loss of the mother or the father^34–36^. Interestingly, the associations between the death of a parent (either the death of the father or the death of the mother) and dementia may be explained by cognitive reserve or brain reserve (i.e., the neural differences in brain size, structure, and number of synapses^28,29^). Indeed, the loss of the father often results in lower socioeconomic status, which then may prevent the building of cognitive reserve^35^. On the other hand, the death of the mother, because she is the main child care provider and has the strongest connection (e.g., biological, behavioral, emotional, and cognitive) with the child, could alter the cognitive development of the child, leading to lower brain reserve and cognitive impairment in late-life^34–36^. Although the study of Norton et al.^35^ demonstrated the importance of the specific adversity experienced (by showing an association between father's death experienced in early life with the increased risk for dementia in late-life but no association for mother’s death at different life course periods), the inconsistency of the results found further highlight—and are mainly explained by—the importance of the period at which the adversity was experienced.

Studies demonstrate that (indicators of) *disadvantageous socioeconomic conditions* or position are associated with lower memory and verbal fluency performance^6,9,18,19,25^. Lower socioeconomic conditions may prevent cognitive reserve to be built up by limiting the access to stimulating activities^27,28^. Disadvantaged socioeconomic conditions may also harm health and impact the building-up of a cognitive reserve through a less beneficial lifestyle^37,38^. Regarding cognitive change and in line with the cognitive reserve hypothesis, disadvantaged socioeconomic conditions are associated with a slower decline in verbal fluency^6^. This has been explained by the fact that the cognitive reserve of the advantaged may compensate for the neuronal loss for a longer period of time but once the underlying pathology becomes substantial, then the speed of decline is faster^29^. However, contrary to this finding stable high life course socioeconomic status has been shown to predict the slowest decline in memory and high late-life income has been found to be the strongest predictor of a slower decline in memory in individuals aged over 50^25^. In addition, it has also been found that socioeconomic position was not related to change in cognition^18^.

Research on *hunger* experienced in childhood shows an association between famine and lower performance on executive function tasks (e.g., Stroop color and word tests) in adulthood, suggesting a long-term effect of hunger on frontal brain regions, but also lower performance in global cognition. Cellular and molecular mechanisms such as alterations in neurotransmitters systems due to malnutrition have been advanced as potential explanations of these results^39^. Another study found that in women not experiencing hunger in childhood is protective (reduced the risk) of global cognitive impairment in older age^40^.

Taken together, similarly to the life course period effect, past research on the effects of specific adversity shows a fragmentary and somewhat contradictory picture. A fundamental limitation of this literature is that it lacks systematic disentangling of the effects of specific adversity from the effects of the life course period at which the adversity was experienced. In fact, several adversities have only been studied at one period of the life course (e.g., death of a parent in childhood and adolescence and hunger in childhood) confounding both effects.

### The present study

Findings on the impact of life course adversity on cognitive performance and on change in cognitive performance in older age seem to differ depending on when adversity is experienced and which specific adversity an individual has been exposed to, as well as the cognitive function investigated. So far, no study has investigated how different adversities experienced at different life course stages affect cognition (both in terms of level and change) within one comprehensive model. The aim of the present study was therefore to investigate the association between life course adversity and cognitive performance (level and change in cognitive performance) in older age by systematically exploring the association of a *comparable set of adversities* on older adults’ cognition depending on when in the life course the specific adversity had been experienced. Based on the literature suggesting particular effects of stress in sensitive periods of early life and late-life on the hippocampus, amygdala, and frontal lobes (regions involved in learning, memory, and higher cognitive functions) and based on the results showing an important relationship between adulthood socioeconomic adversity and cognition, we examined whether at each period of the life course adversity is associated with lower memory and verbal fluency performance in older age and a steeper decline in memory and verbal fluency. We predicted that depending on the life course period at which select adversities are experienced, some adversities will be differentially associated with the level and change in cognitive performance in older age. We expected stress, death of a parent, and hunger experienced in early life and stress in middle adulthood (but not stress in early adulthood and death of a parent experienced in early or middle adulthood) to be negatively associated with cognition (level and change) in older age. We also expected disadvantageous socioeconomic conditions experienced in early life, early and middle adulthood, to be associated with lower level of cognition. In contrast, consistent with the literature suggesting that the early and later life periods are sensitive to stress effects, we expected disadvantageous socioeconomic conditions experienced in early life and in middle adulthood (but not in early adulthood) to be associated with steeper cognitive decline in older age.

## Methods

### Participants

Data of this study stem from the Survey of Health, Ageing and Retirement in Europe (SHARE) database. The first wave of the study started in 2004, and subsequent waves took place every two years until 2017–2018 (for more information on the data collection and more generally on the database, see Börsch-Supan et al.^41^). During waves 1 to 4, SHARE was reviewed and approved by the Ethics Committee of the University of Mannheim. Wave 4 and the continuation of the SHARE project were reviewed and approved by the Ethics Council of the Max Planck Society. All participants provided written informed consent. Only participants over 60 with complete data for cognition across follow-up (wave 1, 2, 4, 5, 6, and 7), without suspicion of dementia in waves 1 and 2 (participants with a score lower than 2 on temporal orientation questions were excluded; see Aartsen et al.^6^; Barbosa et al.^42^, for similar procedures), and having participated to the SHARELIFE retrospective module in waves 3 or 7 were selected. This selection procedure resulted in a final sample of 2′662 participants (*Mdn*_*age*_ = 68, *SD*_*age*_ = 5.39; 1511 (56.76%) of the participants were women). Since cognitive performance improved between the first and the second wave, probably due to learning effects, and was followed by a trend to decline, only waves 2, 4, 5, 6, and 7 were included in our analyses to model cognitive changes over time independently of the probable learning effects occurring between waves 1 and 2.

### Materials

#### Cognition

To measure cognitive performance, delayed recall and verbal fluency performance were used for waves 2, 4, 5, 6, and 7. *Delayed recall* was assessed by recalling a list of 10 words (for more information on the list of words used in the different waves, see SHARE release guide 8.0.0: https://www.share-eric.eu/fileadmin/user_upload/Release_Guides/SHARE_release_guide_8-0-0.pdf). These words were read out loud at an earlier moment, followed by a delay in which the verbal fluency and a numeracy task were completed^43^. Participants received one point for each word correctly recalled after the delay, the total score could thus vary between 0 and 10^44^. The *verbal fluency* task consisted of naming as many different animals as possible in 60 s. The total score was the number of different animals correctly named^43,45^.

#### Adversity

Adversity was assessed via different items matching the adversity definition (i.e., period of stress, hunger, financial hardship experienced, and whether participants had lost their mother and/or father) and that could cover the entire life course (early life, early and middle adulthood). For each of these items, the participants answered yes or no and indicated the year of the beginning of the event. Based on this information, the difference with the date of birth was computed to determine in which life course period the adversity has been experienced. Three life course stages were computed: early life (including adversities experienced up to 20 years old), early adulthood (including adversities experienced from 21 to 40 years old), and middle adulthood (including adversities experienced from 41 to 60 years old).

#### Covariates

Age at the second wave, gender (0 = men, 1 = women), education as well as parental education (coded from 0 = primary to 2 = tertiary education) were used as covariates due to their associations with the variables of interest entered in the model (i.e., adversities and cognition^15,46–51^).

### Statistical analyses

**Figure 1:**
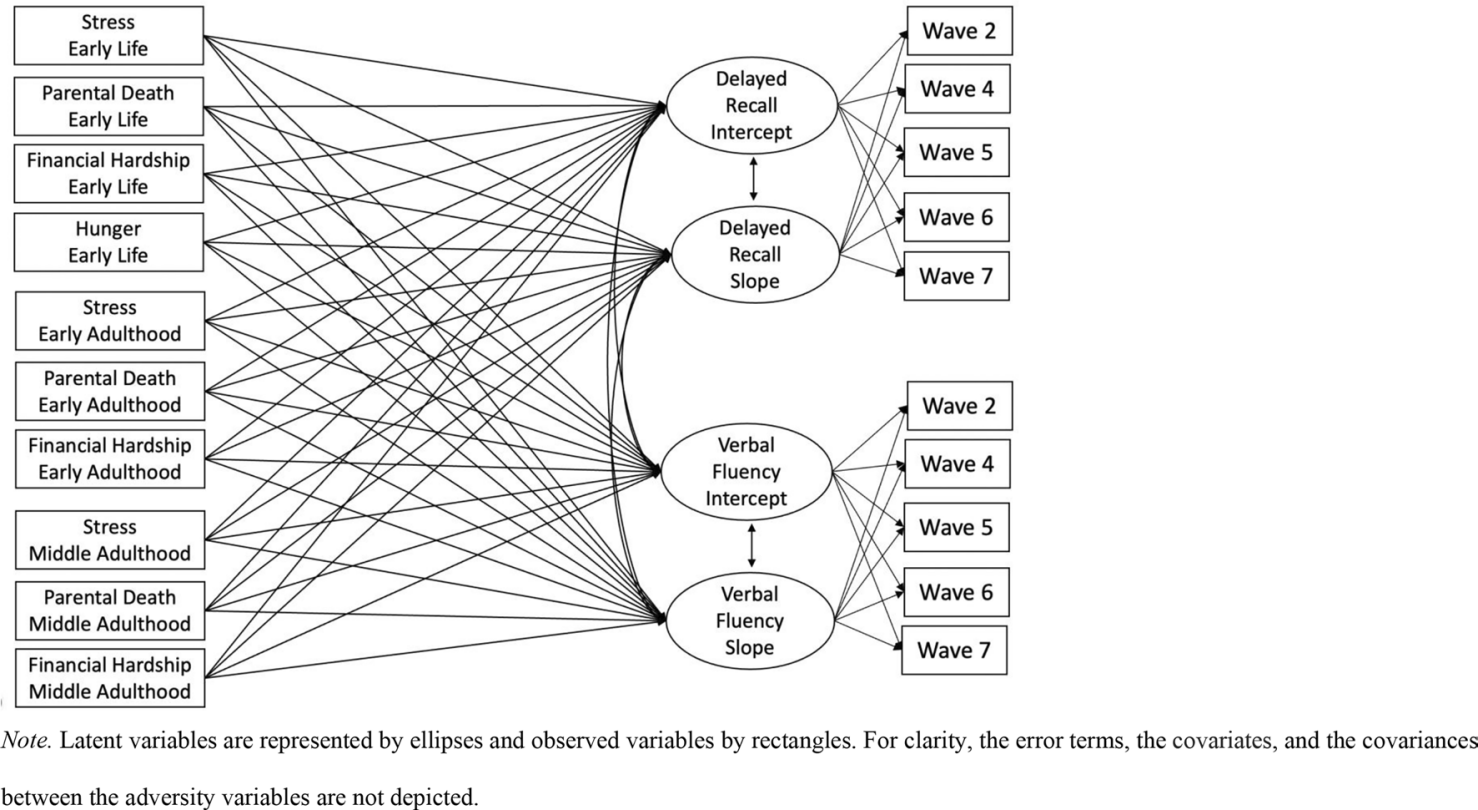
Simplified illustration of the latent growth curve model tested. Latent variables are represented by ellipses and observed variables by rectangles. For clarity, the error terms, the covariates, and the covariances between the adversity variables are not depicted.

Data were analyzed in a structural equation modeling (SEM) framework^52^ (see Fig. 1 for a simplified illustration of the final model). The model’s goodness of fit was assessed using the comparative Fit Index (CFI), considered as good when it is equal to or higher than 0.95, the Root Mean Squared Error of Approximation (RMSEA), considered as good when lower than 0.06, and the Standardized root-mean-square residual (SRMR), considered as good when equal to or lower than 0.08^53,54^.

In a preliminary step, we determined the best fitting slope to model change across waves in both delayed recall and verbal fluency. Accordingly, we fitted three latent growth curve models for each task separately: (1) linear, (2) linear plus quadratic, and (3) freely estimated time scores. Since models with the linear plus quadratic slopes failed to converge, we compared the linear models and freely estimated time scores models using the Satorra-Bentler scaled chi-square difference test^55^, to determine which of them fitted the data better. We accordingly retained the model with the freely estimated time scores both for delayed recall (Δχ^2^_(Δ*df*=3)_ = 26.04, *p* < 0.05) and verbal fluency (Δχ^2^_(Δ*df*=3)_ = 8.44, *p* < 0.05). Note that the four models fitted had satisfactory goodness of fit.

In our final model, we combined the selected latent growth curve models for the two cognitive variables in a single model and included each of the adversity observed variables (stress, parental death, financial hardship, and hunger) of each of the life course stages (except hunger for early adulthood and middle adulthood due to the low occurrence of this event; 1.16% for early adulthood and 0.11% for middle adulthood) as independent predictors of the latent intercepts (i.e., level) and slopes (i.e., change) for the delayed recall and verbal fluency latent variables (see Fig. 1). There was no multicollinearity between the adversity variables and the covariates as indicated by the variance inflation factor (VIF) values lower than 1.79. Since the outcomes observed variables (delayed recall and verbal fluency) deviated from normality, we used maximum likelihood estimation with robust standard errors (MLR) in Mplus (version 8.5^56^). Missing values on the predictors were handled using Full Information Maximum Likelihood (FIML).

## Results

Descriptive statistics for the outcome variables and covariates as well as frequency tables for the adversity indicators are available in Tables 1 and 2 (see also supplements Figs. A and B for the sample means of cognitive performance at each wave). The fit of the model was very good, CFI = 0.98, RMSEA = 0.03, and SRMR = 0.02. The estimated means and variances of the intercepts and slopes for delayed recall and verbal fluency were significant, indicating that participants not only differed in their overall performance in these two cognitive tasks but also in their rate of change over time (see Table 3). Table 4 shows the loading estimates for the freely estimated time scores indicating the form of change in delayed recall and verbal fluency performance across waves.

**Table 1:** Descriptive statistics for the delayed recall and verbal fluency observed variables and for the covariates age, father, mother, and participant’s education.

Variables	*N*	Waves	*M*	Variance	Minimum	Maximum	Percentage with minimum (%)	Percentage with maximum (%)	*Mdn*
Delayed recall	2662	Wave 2	3.67	3.66	0	10	5.90	0.38	4.00
	2662	Wave 4	3.66	4.20	0	10	8.98	0.23	4.00
	2662	Wave 5	3.55	4.48	0	10	10.56	0.34	4.00
	2662	Wave 6	3.44	4.55	0	10	12.36	0.23	3.00
	2662	Wave 7	3.13	4.46	0	10	16.08	0.23	3.00
Verbal fluency	2662	Wave 2	19.97	49.86	0	74	0.04	0.04	19.00
	2662	Wave 4	18.84	45.54	2	93	0.08	0.04	18.00
	2662	Wave 5	19.06	52.34	0	100	0.04	0.04	18.00
	2662	Wave 6	18.69	50.57	0	88	0.04	0.04	18.00
	2662	Wave 7	18.22	60.53	0	100	0.41	0.08	18.00
Age	2662		69.23	29.02	61	90	0.60	0.04	68.00
Father's education	2600		0.48	0.44	0	2	61.62	9.65	0.00
Mother's education	2640		0.34	0.29	0	2	69.89	3.52	0.00
Participant's education	2643		0.84	0.56	0	2	37.31	20.92	1.00

**Table 2:** Frequency table for the adversity variables for each life course stage.

Life course stages	Adversity	*N*	Adversity experienced	
			Yes	No
Early life	Stress	2640	85 (3.22%)	2555 (96.78%)
	Parental death	2501	503 (20.11%)	1998 (79.89%)
	Financial hardship	2642	129 (4.88%)	2513 (95.12%)
	Hunger	2654	264 (9.95%)	2390 (90.05%)
Early adulthood	Stress	2632	589 (22.38%)	2043 (77.62%)
	Parental death	2516	1138 (45.23%)	1378 (54.77%)
	Financial hardship	2632	549 (20.86%)	2083 (79.14%)
Middle adulthood	Stress	2605	895 (34.36%)	1710 (65.64%)
	Parental death	2525	1752 (69.39%)	773 (30.61%)
	Financial hardship	2605	368 (14.13%)	2237 (85.87%)

**Table 3:** Estimated means and variances for the latent intercepts and slopes of delayed recall and verbal fluency.

Outcomes	Latent variables	*M*	*SE*	Variance
Delayed recall	Intercept	3.75***	0.04	2.09***
	Slope	− 0.12***	0.01	0.06***
Verbal fluency	Intercept	19.86***	0.14	31.42***
	Slope	− 0.35***	0.03	0.44***

***p < 0.001.

**Table 4:** Estimated cumulative change for delayed recall and verbal fluency.

Outcomes	Waves	Estimate	*SE*	Standard error/estimate	Fixed effect
Delayed recall	Wave 2	0.00	0.00	999.00	0
	Wave 4	1.28***	0.22	5.79	Free
	Wave 5	2.20***	0.22	9.97	Free
	Wave 6	3.18***	0.23	14.15	Free
	Wave 7	5.00	0.00	999.00	5
Verbal fluency	Wave 2	0.00	0.00	999.00	0
	Wave 4	2.13***	0.25	8.66	Free
	Wave 5	2.52***	0.22	11.39	Free
	Wave 6	3.43***	0.23	14.95	Free
	Wave 7	5.00	0.00	999.00	5

***p < 0.001.

As these are freely estimated time scores, waves 2 and 7 were fixed to 0 and 5 respectively. Delayed recall performance declined quite slowly between waves 2 and 4 (an estimated mean change of 1.28 in delayed recall in 4 years), followed by a more rapid decline between waves 4 and 6 (with an estimated mean change of 0.92 in 2 years between wave 4 and 5, and 0.98 between wave 5 and 6, thus a decline of 1.90 in 4 years), then this decline became even steeper between waves 6 and 7 (an estimated mean change of 1.82 in 2 years between waves 6 and 7).

Verbal fluency performance declined quite rapidly between waves 2 and 4 (an estimated mean change of 2.13 in verbal fluency in 4 years), followed by a slight decline between waves 4 and 5 (an estimated mean change of 0.39 in 2 years) and a more rapid decline between waves 5 and 6 (an estimated mean change of 0.91 in 2 years). Finally, this decline became even steeper between waves 6 and 7 (an estimated mean change of 1.57 in 2 years).

### Level of delayed recall performance

Hunger (β = − 0.10, *p* < 0.001) experienced in early life, predicted lower delayed recall performance in older age. Effects of stress, parental death, and financial hardship in early life were not significant (see Table 5 and supplements Fig. C).

**Table 5:** Standardized estimates for regression weights of life course adversity on the level (intercept) and non-linear change (slope) of delayed recall and verbal fluency and explained variances (R-square) of the intercepts and slopes of delayed recall and verbal fluency.

Life course stages	Adversity	Delayed recall		Verbal fluency	
		Level	Non-linear change	Level	Non-linear change
Early life	Stress	0.01	0.02	0.00	0.01
	Death of a parent	− 0.03	0.01	− 0.01	0.003
	Financial hardship	− 0.01	− 0.11***	− 0.06**	0.04
	Hunger	− 0.10***	0.05	− 0.06***	0.01
Early adulthood	Stress	0.07**	0.03	0.10***	− 0.04
	Death of a parent	0.02	− 0.03	− 0.01	0.09
	Financial hardship	0.08***	− 0.02	0.08***	− 0.04
Middle adulthood	Stress	0.04	− 0.004	0.03	0.01
	Death of a parent	0.04	− 0.05	0.003	0.02
	Financial hardship	− 0.04	0.07*	− 0.03	0.01
Covariates	Age	− 0.15***	− 0.23***	− 0.09***	− 0.24***
	Gender	0.31***	− 0.06	− 0.06	− 0.002
	Father's education	0.12***	− 0.07	0.10***	− 0.03
	Mother's education	0.05	0.02	0.07**	0.05
	Education	0.40***	0.04	0.40***	− 0.03
R-square of latent variables		0.36***	0.08***	0.31***	0.07**

*p ≤ 0.05. **p ≤ 0.01. ***p ≤ 0.001.

Stress (β = 0.07, *p* = 0.003) and financial hardship (β = 0.08, *p* = 0.001) experienced in early adulthood predicted better delayed recall performance in older age. Effect of parental death in early adulthood was not significant (see Table 5 and supplements Fig. C).

None of the adversities experienced in middle adulthood were significant predictors of delayed recall performance in older age (see Table 5 and supplements Fig. C).

Age (β = − 0.15, *p* < 0.001), gender (β = 0.31, *p* < 0.001), father’s education (β = 0.12, *p* < 0.001), and participant’s education (β = 0.40, *p* < 0.001) significantly predicted delayed recall performance in older age, such that older participants, men, those whose fathers had lower education and those being less educated had worse delayed recall performance. The effect of mother’s education did not reach significance (see Table 5).

Together the predictors explained a significant 36% of the delayed recall performance (see Table 5).

### Level of verbal fluency performance

Financial hardship (β = − 0.06, *p* = 0.005) and hunger (β = − 0.06, *p* = 0.001) experienced in early life predicted lower verbal fluency performance in older age. Effects of stress and parental death in early life were not significant (see Table 5 and supplements Fig. C).

Stress (β = 0.10, *p* < 0.001) and financial hardship (β = 0.08, *p* = 0.001) experienced in early adulthood predicted better verbal fluency performance in older age. Effect of parental death in early adulthood was not significant (see Table 5 and supplements Fig. C).

None of the adversities experienced in middle adulthood were significant predictors of verbal fluency performance in older age (see Table 5 and supplements Fig. C).

Age (β = − 0.09, *p* < 0.001), father’s education (β = 0.10, *p* < 0.001), mother’s education (β = 0.07, *p* = 0.008), and participant’s education (β = 0.40, *p* < 0.001) significantly predicted verbal fluency performance in older age, such that older participants, those whose fathers and mothers had lower education and those being less educated had worse verbal fluency performance. The effect of gender did not reach significance (see Table 5).

Together the predictors explained a significant 31% of the verbal fluency performance (see Table 5).

### Non-linear change in delayed recall performance

Financial hardship (β = − 0.11, *p* < 0.001) experienced in early life predicted a steeper decline in delayed recall performance across waves. Effects of stress, parental death, and hunger in early life were not significant (see Table 5 and supplements Fig. C).

None of the adversities experienced in early adulthood were significant predictors of non-linear change in delayed recall across waves (see Table 5 and supplements Fig. C).

Financial hardship (β = 0.07, *p* = 0.048) experienced in middle adulthood predicted a lower decline in delayed recall performance across waves. Effects of stress and parental death in late adulthood were not significant (see Table 5 and supplements Fig. C).

Age (β = − 0.23, *p* < 0.001) significantly predicted a steeper decline in delayed recall performance in older age, such that older participants, declined more steeply in delayed recall performance across waves. The effect of gender, father and mother’s education as well as participant’s education did not reach significance (see Table 5).

Together the predictors explained a significant 8% of the change in delayed recall performance across waves (see Table 5).

### Non-linear change in verbal fluency performance

None of the adversities experienced in early life, early adulthood, and middle adulthood were significant predictors of non-linear change in verbal fluency performance across waves (see Table 5 and supplements Fig. C).

Age (β = − 0.24, *p* < 0.001) significantly predicted a steeper decline in verbal fluency performance across waves, such that older participants had a steeper decline in verbal fluency performance. The effect of gender, father and mother’s education as well as participant’s education did not reach significance (see Table 5).

Together the predictors explained a significant 7% of the change in verbal fluency performance across waves (see Table 5).

## Discussion

The present study set out to examine the role that different life course periods play in the association of distinct adverse experiences with individual differences in cognitive aging: Does the life period at which the adversity was experienced matter and if so does it matter particularly for some but not other adverse experiences? Taken together, the present analyses suggest that *both the period* of adverse experiences and *specific* adversity matter, as only specific adversities experienced at a specific life course period negatively predicted delayed recall and verbal fluency level of performance and change in performance.

In detail, we found that the *early experience of financial hardship* predicted lower verbal fluency performance, and the experience of *hunger in early life* predicted lower delayed recall as well as lower verbal fluency performance. This result is consistent with research stating that early life adversity has long-lasting impacts on brain and cognition in older age compared to adulthood^12,14,17,57^. This finding is also in line with studies suggesting a long-term effect of hunger and disadvantageous socioeconomic conditions experienced in early life on cognition^6,19,39^. Moreover, the experience of *financial hardship in early life* predicted a *steeper decline* in delayed recall highlighting that—contrary to recent arguments regarding education that only seems to affect the level and not the change in cognition in aging^49,58^—*financial hardship* experienced in *early life* seems to influence *both* the *level* in verbal fluency performance and *change* in delayed recall. This negative association between the financial hardship experienced in early life and both level and change in cognition may be explained by financial hardship in early life leading to less stimulations (mental stimulation and stimulating activities) and worse lifestyle^6,37,38^. As a consequence lower level of cognitive reserve may be built up as opposed to individuals that do not experience this adversity^28,29^. Regarding hunger, the exposure to hunger may cause alterations in neurotransmitters systems impacting cognition^39^ which may, therefore, explain the negative association found between hunger in early life and the level of cognition. Further research is needed to target those potential pathways directly.

We also found that adversities experienced *later in life* (in early and middle adulthood) *did not negatively predict* cognition and change in cognition in older age. On the contrary, *stress and financial hardship* experienced in *early adulthood predicted better* delayed recall and verbal fluency performance in older age, and *financial hardship experienced in middle adulthood* predicted a *lower decline* in delayed recall. The evidence that stress and financial hardship experienced in early adulthood were positively related to the level of cognition—although counterintuitive at first view—is in line with the study of Rosnick et al.^10^ showing a positive association between some adversity (i.e., illness or injury of a friend) with episodic memory performance. However, in general, this result contradicts previous literature showing a negative association between one type of adulthood adversity (i.e., socioeconomic conditions) and memory (i.e., composite score in memory and prospective memory) in late-life^9,25^. While being speculative at this point, the associations found may be explained as a result of the experience of using effective coping processes, since previous experiences of adversity could foster resilience under some circumstances^59^. Nevertheless, for the period of stress, as the stressor was not identified, one cannot know whether the stressor and thus the period of stress was an adversity in itself. The finding that financial hardship experienced in middle adulthood led to less decline in delayed recall is in line with the results of a study on adults aged between 55 and 85 years old, showing that mild ongoing chronic stressors were associated with less decline in global cognition in older adults^7^. One explanation may be that a moderate level of stress induced by an ongoing stressor can enhance cognitive performance, in particular memory (inverted-U shape function between glucocorticoids and cognitive performance^33^). Another explanation might be that financial hardship in middle adulthood may lead individuals to actively engage in paid work after retirement, requiring them to be involved in cognitively stimulating activities or being in a stimulating environment (cf. cognitive reserve hypothesis), and thus may explain this lower decline in delayed recall performance^60^. Again, further research to follow up on those findings is needed.

Finally, our results *positively* relate stress and financial hardship in early adulthood to cognition, and show a lower decline in delayed recall for individuals experiencing financial hardship in middle adulthood, this nicely dovetails with the cognitive reserve hypothesis. Indeed, in early and middle adulthood, cognitive reserve is already well accumulated compared to early life where the cognitive reserve is still being accrued via education, the first occupation, and stimulating leisure activities. Hence, the exposure to adversity in early life may hinder the major accumulation processes of cognitive reserve, which may be less important for later phases in life^26,28,29^. Moreover, individuals in adulthood may have accumulated critical resources to cope with adversity (or may have more experiences with adversities) and may better use them compared to resources available in early life^59,61^. Besides, stress and financial hardship experienced in adulthood (early or middle) may be actively coped with increasing creativity, compared to adversity experienced in early life where the children have less control over the situation and mainly depend on their parents. Indeed, this seems to be the case for people with a high resilience trait where a positive relationship between post-traumatic stress symptoms and creative thinking has been found^62^.

Contrary to some studies that have reported an association between early parental death and cognition, we found that parental death had no significant effect on cognition (level and change in cognition) at any period of the life course^34–36^. This result may be explained by the more fine gradient of the early life category used in the studies cited. Indeed, most of these studies divide the early category into two categories (childhood parental death and parental death in adolescence) and separate the effect of father’s death from the effect of mother’s death. Hence, our category being broader than the ones in the previous studies and the death of the mother and father being aggregated, the negative association between parental death (father or mother) in childhood or adolescence and cognition may be suppressed by the non-effect of parental death experienced in late-adolescence or by the lack of distinction between father and mother’s death. Moreover, the present study does not take into account whether or not the widowed parent was remarried which seems to diminish the relationship between parental death and the rate of Alzheimer’s disease in older age^36^. However, our results tend to follow those of Norton et al.^35^, by not finding significant associations between parental death later in life and cognition in older age.

### Strength and limitations of the research

The present study has the advantage to use a *large population-based database* of non-institutionalized older people. Moreover, this dataset has a *substantial longitudinal follow-up* from 2006 to 2017–2018, allowing to have more than the four waves necessary to estimate non-linear changes using Latent Growth Curve Modeling. The dataset included data on diverse adversities, information when the adversity has been experienced, as well as repeated assessments of different cognitive functions (memory with delayed recall and executive functions with verbal fluency). Hence, in addition to studying the association between a specific adversity and the level of cognition (delayed recall and verbal fluency), this study investigated the association between a set of adversities (stress, parental death, financial hardship, and hunger) experienced at different life course periods and the level of cognition as well as the change in cognitive performance across five measurement occasions. In addition, the period-of-stress item—in contrast to other items in the model—allows us to gain broader information about the subjective experience of stress during a certain period independently of the specific stressor that has been experienced. Importantly, this study considered different adversities without aggregating them while still controlling for their dependencies (see Table A in supplements for standardized estimates for covariance between the adversity variables).

In terms of limitations, we acknowledge that adversity experiences as well as the period at which the adversity had been experienced were retrospectively reported and thus subject to reporting biases. Therefore, the influence of the participant’s level of cognitive performance and/or cognitive preservation on the reporting of adversity and the period of the adverse event cannot be excluded. Moreover, due to our analytical design crossing specific adversities with life course periods, only a selection of life course adversities (those mainly present in each of the life course periods) was examined. Besides, as we have no information on the perception of adversity (subjective experience) nor the stressor causing the stress period, we cannot affirm that at the individual level these events, life conditions, and the period of stress were experienced aversively. Regarding the cognitive tests, further learning effects beyond wave 2 cannot be excluded. Since only participants over 60 who have participated in all 6 waves assessing cognition were selected, a survivor, as well as a selection bias^63,64^, may be present and explain some of the results found, specifically a health selection bias^65–68^ and a resilience selection bias.

### Outlook

In future studies, alternative tasks to assess cognitive performance longitudinally should be used to avoid learning effects. In addition, the experience of adversity is subjective and individuals diverge not only in the appraisal of the event as being adverse but also in the capacity to cope with the adverse event. Future research should include more information related to the adversity experienced, social support, and resilience. For example, in assessing the appraisal of the adversity experienced, asking the level of stress related to this adversity experienced, and distinguishing between the severity as well as the duration, the chronicity of the adverse event experienced, and collecting information on the social support and the capacity to cope with the adverse event throughout the life course. Finally, studies should also consider the genetic, epigenetic, and biological, factors involved in the relationship between adversity and cognition, taking into account their interactions (over time) and the timing of exposure to adversity^1,4,7,69–71^. The aim would be to gain a more fine gradient on the effect of different types of adversity at different life course stages on cognition and change in cognition as well as the factors and mechanisms playing a role in these associations.

## Conclusion

In conclusion, this study found that adversity experienced in early life (and here, especially hunger and financial hardship) was negatively related to cognitive aging which was not the case of adversity experienced later in life, suggesting the importance of the sensitive period (early life) in the experience of adversity. Conceptually, the results of this study underline the importance of disentangling adversities and sub-domains of socioeconomic conditions as—in contrast to education—financial hardship experienced in early life did indeed affect *cognitive change* in aging. Moreover, the differential results for memory and verbal fluency measures suggest that is also important to consider the cognitive outcome domains examined when investigating the relationship between life course adversity and late-life cognition. The findings of the present study highlighting important adverse effects of financial hardship and hunger early in life on later life cognitive health are especially relevant from a social policy perspective.

## Supplementary Information

Supplementary Information.

## Data Availability

Data are available in open access (http://www.share-project.org/data-access.html). This study was not preregistered.
