# Supplementary material for: Adversity specificity and life period exposure on cognitive aging
Source: Sci Rep. 2023 May 29;13:8702. doi: 10.1038/s41598-023-35855-5 (PMC10227009; doi:10.1038/s41598-023-35855-5)

**Figure A**

Delayed Recall Sample Means for Each Wave of Interest

**
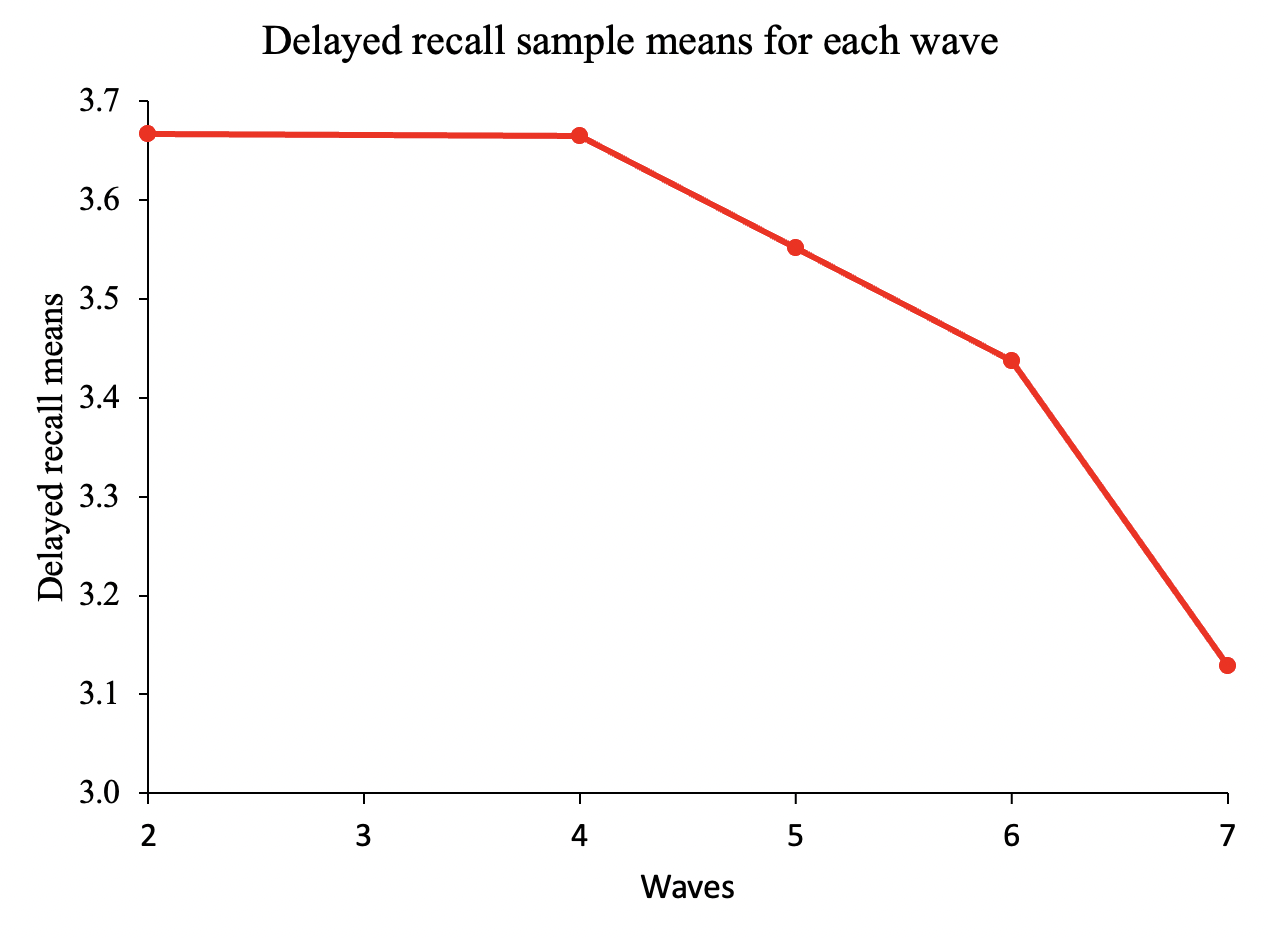
**

**Figure B**

Verbal Fluency Sample Means for Each Wave of Interest


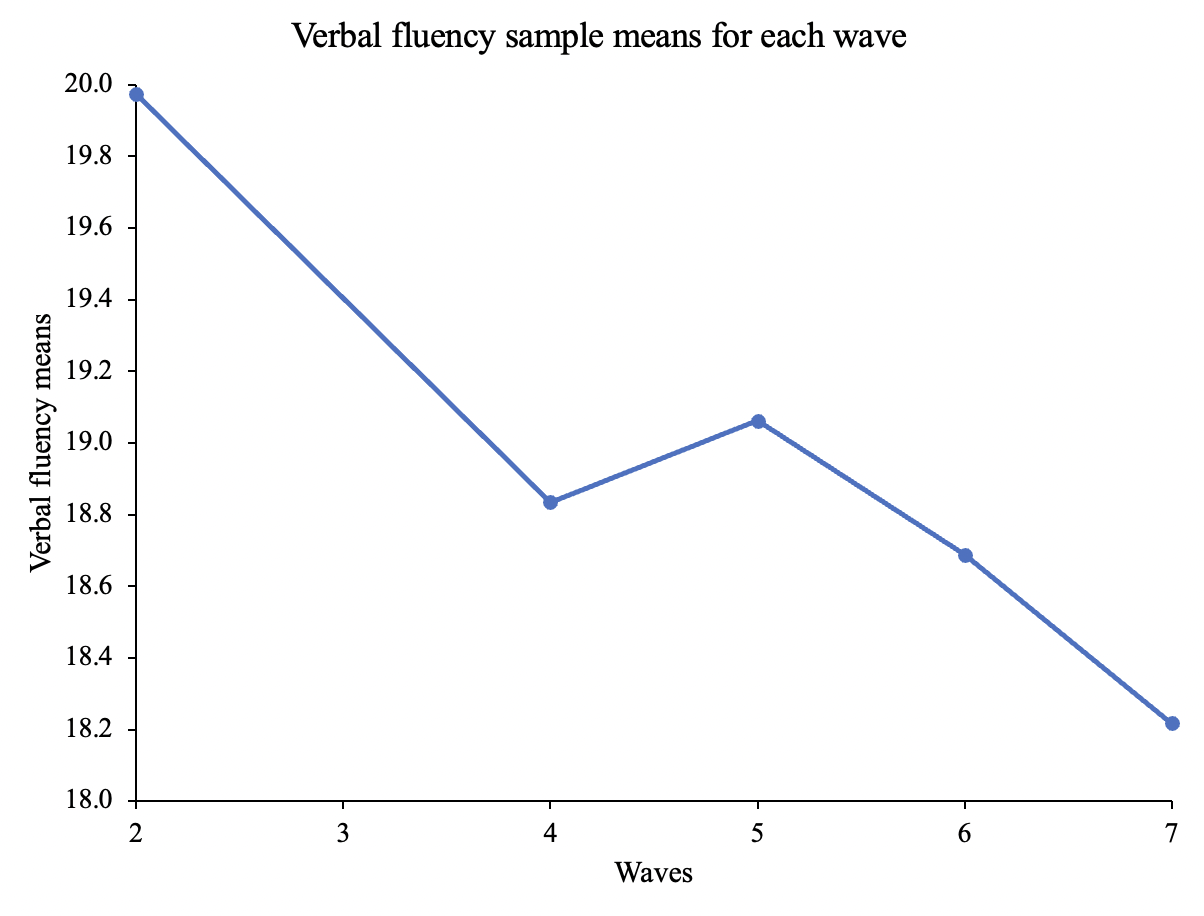


**Table A**

*Standardized Estimates for Covariance between the Adversity Variables*

| Adversity variables | | Standardized estimates for covariance | *p*-value |
| --- | --- | --- | --- |
| Stress early life |  |  |  |
|  | Parental death early life | .04 | .11 |
|  | Financial hardship early life | .14 | <.001 |
|  | Hunger early life | .07 | .01 |
|  | Stress early adulthood | .22 | <.001 |
|  | Parental death early adulthood | -.004 | .85 |
|  | Financial hardship early adulthood | .09 | <.001 |
|  | Stress middle adulthood | -.06 | .001 |
|  | Parental death middle adulthood | -.05 | .02 |
|  | Financial hardship middle adulthood | .03 | .25 |
| Parental death early life |  |  |  |
|  | Financial hardship early life | .06 | .01 |
|  | Hunger early life | .06 | .01 |
|  | Stress early adulthood | -.04 | .04 |
|  | Parental death early adulthood | -.19 | <.001 |
|  | Financial hardship early adulthood | .05 | .02 |
|  | Stress middle adulthood | -.02 | .26 |
|  | Parental death middle adulthood | -.25 | <.001 |
|  | Financial hardship middle adulthood | -.02 | .28 |
| Financial hardship early life |  |  |  |
|  | Hunger early life | .18 | <.001 |
|  | Stress early adulthood | .04 | .046 |
|  | Parental death early adulthood | -.03 | .09 |
|  | Financial hardship early adulthood | .27 | <.001 |
|  | Stress middle adulthood | -.02 | .42 |
|  | Parental death middle adulthood | -.003 | .87 |
|  | Financial hardship middle adulthood | -.01 | .72 |
| Hunger early life |  |  |  |
|  | Stress early adulthood | .02 | .34 |
|  | Parental death early adulthood | -.02 | .40 |
|  | Financial hardship early adulthood | .10 | <.001 |
|  | Stress middle adulthood | .02 | .28 |
|  | Parental death middle adulthood | -.04 | .053 |
|  | Financial hardship middle adulthood | -.001 | .96 |
| Stress early adulthood |  |  |  |
|  | Parental death early adulthood | .01 | .71 |
|  | Financial hardship early adulthood | .17 | <.001 |
|  | Stress middle adulthood | .17 | <.001 |
|  | Parental death middle adulthood | -.002 | .91 |
|  | Financial hardship middle adulthood | .06 | .004 |
| Parental death early adulthood |  |  |  |
|  | Financial hardship early adulthood | -.01 | .66 |
|  | Stress middle adulthood | -.03 | .09 |
|  | Parental death middle adulthood | -.33 | <.001 |
|  | Financial hardship middle adulthood | .02 | .44 |
| Financial hardship early adulthood |  |  |  |
|  | Stress middle adulthood | .08 | <.001 |
|  | Parental death middle adulthood | -.04 | .03 |
|  | Financial hardship middle adulthood | .24 | <.001 |
| Stress middle adulthood |  |  |  |
|  | Parental death middle adulthood | .03 | .11 |
|  | Financial hardship middle adulthood | .17 | <.001 |
| Parental death middle adulthood |  |  |  |
|  | Financial hardship middle adulthood | .001 | .94 |

**Figure C**

*Summary of the Significant Associations Found between Experiencing Adversity and the Level and Change in Cognitive Performance*


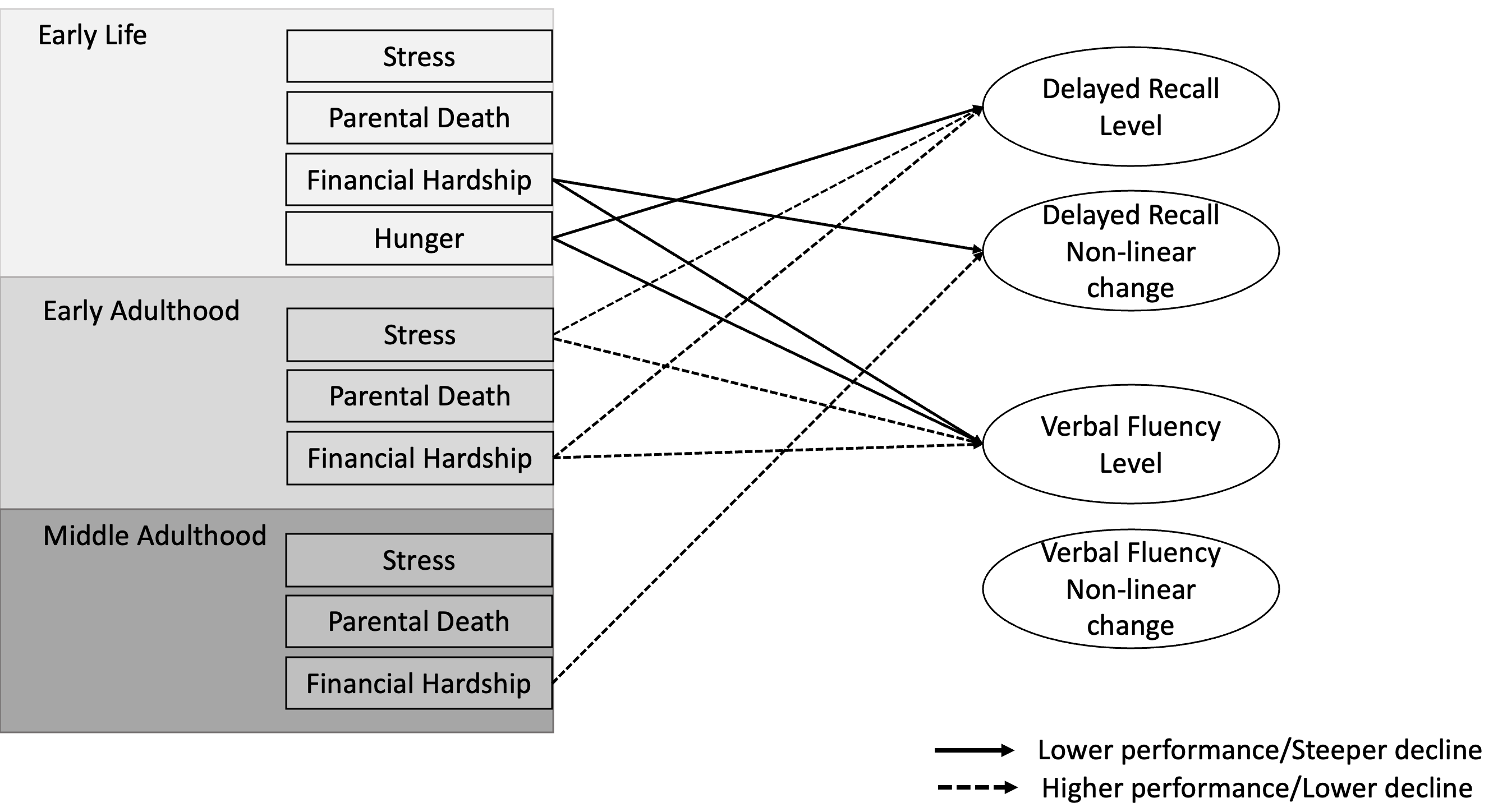

Supplement: Supplementary file 1 — Supplementary Information. [file 41598_2023_35855_MOESM1_ESM.docx]
